# Subclinical Inflammatory Status in Rett Syndrome

**DOI:** 10.1155/2014/480980

**Published:** 2014-01-06

**Authors:** Alessio Cortelazzo, Claudio De Felice, Roberto Guerranti, Cinzia Signorini, Silvia Leoncini, Alessandra Pecorelli, Gloria Zollo, Claudia Landi, Giuseppe Valacchi, Lucia Ciccoli, Luca Bini, Joussef Hayek

**Affiliations:** ^1^Child Neuropsychiatry Unit, University Hospital Azienda Ospedaliera Universitaria Senese (AOUS), Viale M. Bracci 16, 53100 Siena, Italy; ^2^Department of Medical Biotechnologies, University of Siena, Via A. Moro 2, 53100 Siena, Italy; ^3^Neonatal Intensive Care Unit, University Hospital AOUS, Viale M. Bracci 16, 53100 Siena, Italy; ^4^Department of Molecular and Developmental Medicine, University of Siena, Via A. Moro 6, 53100 Siena, Italy; ^5^Department of Life Science, University of Siena, Via A. Moro 2, 53100 Siena, Italy; ^6^Department of Life Sciences and Biotechnology, University of Ferrara, Via Borsari 46, 44100 Ferrara, Italy; ^7^Department of Food and Nutrition, Kyung Hee University, 1 Hoegi-dong, Dongdaemun-gu, Seoul 130-701, Republic of Korea

## Abstract

Inflammation has been advocated as a possible common central mechanism for developmental cognitive impairment. Rett syndrome (RTT) is a devastating neurodevelopmental disorder, mainly caused by *de novo* loss-of-function mutations in the gene encoding MeCP2. Here, we investigated plasma acute phase response (APR) in stage II (i.e., “pseudo-autistic”) RTT patients by routine haematology/clinical chemistry and proteomic 2-DE/MALDI-TOF analyses as a function of four major *MECP2* gene mutation types (R306C, T158M, R168X, and large deletions). Elevated erythrocyte sedimentation rate values (median 33.0 mm/h versus 8.0 mm/h, *P* < 0.0001) were detectable in RTT, whereas C-reactive protein levels were unchanged (*P* = 0.63). The 2-DE analysis identified significant changes for a total of 17 proteins, the majority of which were categorized as APR proteins, either positive (*n* = 6 spots) or negative (*n* = 9 spots), and to a lesser extent as proteins involved in the immune system (*n* = 2 spots), with some proteins having overlapping functions on metabolism (*n* = 7 spots). The number of protein changes was proportional to the severity of the mutation. Our findings reveal for the first time the presence of a subclinical chronic inflammatory status related to the “pseudo-autistic” phase of RTT, which is related to the severity carried by the *MECP2* gene mutation.

## 1. Introduction

RTT (OMIM ID: 312750) occurs with a frequency of up to 1 : 10,000 live female births. Causative mutations in the X linked methyl-CpG binding protein 2 gene (*MECP2*) are detectable in up to 95% of cases, although a wide genetical and phenotypical heterogeneity is well established [1]. Approximately 80% of RTT clinical cases show the so-called “typical” clinical picture; after an apparently normal development for 6–18 months, RTT girls lose their acquired cognitive, social, and motor skills in a typical 4-stage neurological regression [2]. It has become apparent that there is a spectrum of severity in RTT, as some patients may present with atypical features, sometimes overlapping with those suggestive of autism spectrum disorders (ASDs) [3–5]. Autistic features are typically transient in RTT, although this condition has long been considered as a genetic/epigenetic model of ASDs [6, 7], RTT has been recently distinct from the ASDs group [8, 9]. Recently, the gene sequence analysis indicates that several hundreds of gene mutations appear to be associated with the *MECP2* gene mutation and, therefore, are to be considered as potential disease modifiers [10], although the genetic mechanisms of RTT have been explored to an extraordinary extent, to date the details of the biological mechanisms linking the *MECP2* gene mutation to protein expression as a function of clinical phenotype, and yet to be clarified. In particular, with the single exception of a proteomic study on a mouse model [11], very little information exists on possible RTT-related protein changes. Although the neuropathology of RTT is well understood, the cellular and molecular mechanisms, leading to the disease initiation and progression, have yet to be elucidated.

Several lines of evidence indicate the presence of an early immune activation in ASDs with an associated peripheral and central chronic inflammation [12–16], with a particular focus on mast cells dysfunction and cytokines dysregulation [12, 14].

To date, experimental and clinical evidence has generated the idea that several serum proteins, considered as biomarkers, are strictly correlated with the pathophysiology of the autistic disorder [17–20]. In particular, significant changes in inflammation-related proteins suggested that at least some autistic children display a subclinical inflammatory state [21]. During inflammation, particularly during the APR, there is a known reduction in several proteins potentially affecting cholesterol transport and inhibiting oxidation phenomena. This protein list includes cholesterol ester transfer protein, hepatic lipase, and apolipoproteins. It is thought that reduction in these proteins, associated with an increase in positive APR proteins, may change the high density lipoprotein from anti-inflammatory into proinflammatory particles [22]. In the present study, we investigated the occurrence of a plasma APR in stage II (i.e., “pseudo-autistic”) RTT patients by using routine haematology/clinical chemistry and proteomic 2-DE/MALDI-TOF analyses.

## 2. Materials and Methods

### 2.1. Subjects

The study included 25 female patients with clinical diagnosis of typical RTT (median age: 5.0 years inter-quartile range 3–6, values range 3–10 years) with demonstrated *MECP2* gene mutation (R306C (*n* = 5), T158M (*n* = 5), R168X (*n* = 8), and large deletions (deletions of exons 3 and 4, *n* = 7)) carrying different phenotype severity, and 40 age-matched healthy controls (median age: 5.0 years inter-quartile range 3–5.5, values range 3–10 years). RTT diagnosis and inclusion/exclusion criteria were based on the recently revised RTT nomenclature consensus [23]. Given the specific aims of the study, subjects with clinically evident inflammatory conditions either acute or chronic were excluded, as well as individuals on anti-inflammatory drugs, or undergoing supplementation with known antioxidants, such as *ω*-3 polyunsaturated fatty acids. All the patients were consecutively admitted to the Rett Syndrome National Reference Centre of the University Hospital of the Azienda Ospedaliera Universitaria Senese (AOUS). The subjects examined in this study were on a typical Mediterranean diet. RTT clinical severity was assessed using either the total clinical severity score (CSS), a validated clinical rating specifically designed for RTT, or the “compressed” CSS both based on 13 individual ordinal categories measuring clinical features common in RTT [24], and phenotypical severity was also measured by the use of the Rett Syndrome Behaviour Questionnaire (RSBQ) [25]. Blood samplings in the patients' group were performed during the routine follow-up study at hospital admission, while the samples from the control group were carried out during routine health checks, sports, or blood donations, obtained during the periodic clinical checks. The healthy control subjects were gender (given that over 98% of RTT patients are females, we selected a female control group) and age-matched. The study was conducted with the approval by the Institutional Review Board and all informed consents were obtained from either the parents or the legal tutors of the enrolled patients.

### 2.2. Sample Collection and Preparation

All samplings from RTT patients and healthy controls were carried out around 8 a.m. after overnight fasting. Blood was collected in heparinized tubes and all manipulations were carried out within 2 h after sample collection.

The blood samples were centrifuged at 2400 g for 15 min at 4°C; the platelet poor plasma was saved and the buffy coat was removed by aspiration. Plasma samples were stored at −70°C until assay.

### 2.3. Erythrocyte Sedimentation Rate (ESR)

The TEST 1 analyzer, a closed automated analyzer, determines the length of sedimentation reaction in blood in a standard-size primary tube with a perforating stopper. The principle of measurement is the study of the aggregation capacity of red blood cells (RBCs) by telemetry. The tubes are placed in appropriate racks, and their contents are rotated slowly for about 2 min. The sample loader simultaneously accepts 4 racks containing 15 tubes each. By using a closed aspiration needle, the blood is directly drawn from the collection tube, distributed in a capillary, and centrifuged at about 20 g. The sensing area temperature is maintained at 37°C. The system uses an infrared ray microphotometer with a light wavelength of 950 nm and performs 1,000 readings during 20 seconds. The electrical impulses, collected using a photodiode detector, are directly correlated to the aggregation of RBCs present at each capillary level. For each sample, an aggregation and sedimentation curve is obtained. A mathematical algorithm converts the raw data obtained from evaluation of optical density signals into ESR results, which are transformed to comparable Westergren values. The system operates at a rate of 180 specimens per hour in continuous loading, providing a result every 20 seconds, and requires 150 *μ*L of blood for each sample [26, 27].

### 2.4. C-Reactive Protein (CRP)

A Modular analytics P module (Roche, Hitachi) was used to determine serum CRP by immunoturbidimetry using 3rd generation CRP Tina-quant reagent (Roche, Hitachi). The functional sensitivity for CRP testing was 0.042 mg/dL. The interval reference was <0.5 mg/dL for CRP [28].

### 2.5. Two-Dimensional Gel Electrophoresis (2-DE)

2-DE is a protein separation technique that combines two different electrophoretic methods: isoelectric focusing (IEF) in the first dimension, in which proteins are separated according to their isoelectric points (pI), and sodium dodecyl sulfate-polyacrylamide gel electrophoresis (SDS-PAGE) in the second dimension, in which proteins are separated according to their molecular weights (MW) [29]. Samples containing 60 *μ*g of protein as determined by Bradford [30] were denatured with 10 mL of a solution containing 10% of SDS and 2.3% of dithiothreitol (DTT). Afterwards, samples were combined with 350 mL of solubilizing buffer containing 8 M urea, 2% of 3-[(3-cholamidopropyl)-dimethylammonium]-1-propane sulfonate (CHAPS), 0.3% DTT, and 2% of immobilized pH gradient (IPG) buffer, and loaded into 18 cm IPG strips 3–10 nonlinear on an Ettan IPGphor (GE Healthcare) apparatus system, and rehydrated for 7 h. IEF was carried out for a total of 32 kV h. After focusing, the strips were equilibrated with the buffer containing 50 mM Tris-HCl, pH 8.8, 6 M urea, 2% w/v SDS, 30% v/v glycerol, and 1% w/v DTT for 15 min. Subsequently, strips were equilibrated again with the same equilibration buffer described above, except that it contained 4% w/v iodoacetamide instead of DTT and a trace of bromophenol blue. The second dimension was performed on an EttanDalt Six Electrophoresis system (GE Healthcare). IPG strips and a MW standard were embedded at the top of a 1.5 mm thick vertical polyacrylamide gradient gel (8–16% T) using 0.5% w/v agarose and run at a constant current of 40 mA/gel at 20°C. Each sample was carried out in triplicate under the same conditions.

### 2.6. Image Analysis

Images of gels were analyzed using ImageMaster 2D Platinum v7.0 software (GE Healthcare). The reference gel for each group (i.e., healthy controls, RTT, R306C, T158M, R168X, and large deletions in exons 3 and 4) was defined and used for the comparative analyses. The background was subtracted from all gels using the average-on-boundary method. Spot volume was expressed as a ratio of the total protein percentage volume (%V) detected from the entire gel to minimize differences between gels (gel normalization), for pooling them. Only spots appearing in all gels of the same group were matched with those of the reference gel.

### 2.7. Trypsin Digestion and Proteins Identification by Mass Spectrometry

2-DE/MALDI-TOF is a complex and advanced technique to identify and characterize proteins from biological fluids and/or tissues whose results are considered to be reliable and reproducible when fitting a series of different parameters, such as peptide matches, sequence coverage (%), MOWSE score, and pI/relative molecular mass (Mr, kDa). A 2-DE/MALDI-TOF approach is helpful in order to reveal global protein pattern changes in a given tissue/body fluid for a given condition. However, the technique has assay sensitivity limitations when applied to either less abundant or small proteins (i.e., cytokines) [22, 31].

After mass spectrometry compatible silver staining, the preparative gel was matched to the master gel in the analytical gel match set [32]. A spot-picking list was generated and exported to Ettan Spot Picker (GE Healthcare). The spots were excised and delivered into 96-well microplates where they were destained and dehydrated with acetonitrile (ACN) for subsequent rehydration with trypsin solution. Tryptic digestion was carried out overnight at 37°C. Each protein spot digest (0.75 mL) was spotted into the MALDI instrument target and allowed to dry. Then 0.75 mL of the instrument matrix solution (saturated solution of *α*-cyano-4-hydroxycinnamic acid in 50% ACN and 0.5% v/v trifluoroacetic acid) was applied to dried samples and dried again. Mass spectra were obtained, as described in [33], using an ultrafleXtreme MALDI-ToF/ToF (Bruker Corporation, Billerica, MA, USA). After tryptic peptide mass acquisition, mass fingerprint searching was carried out in Swiss-Prot/TREMBL and NCBInr databases using MASCOT (Matrix Science, London, UK, http://www.matrixscience.com). A mass tolerance of 100 ppm was allowed and only one missed cleavage site was accepted. Alkylation of cysteine by carbamidomethylation was assumed as a fixed modification, whereas oxidation of methionine was considered a possible modification. The criteria used to accept identifications included the extent of sequence coverage, number of matched peptides, and probabilistic score.

### 2.8. STRING 9.0 Network Analysis

Possible connections among identified plasma proteins with significant variations as compared to healthy controls expression levels were analyzed by a protein and gene network software. For each protein, UniProtKB entry numbers and related gene names were acquired in UniProtKB and used for network generation by the use of STRING 9.0 (http://www.string-db.org/) [34]. The UniProtKB entry numbers were inserted into the input form as “multiple proteins” and “Homo sapiens” was selected as the reference organism.

### 2.9. Statistical Data Analysis

All variables were tested for normal distribution (D'Agostino-Pearson test) and data were presented as means ± SD for normally distributed variables. Statistical analysis for protein expressed differently in the groups was carried out using GraphPad Prism software using Student's *t*-test and one-way ANOVA test. Bonferroni-corrected significance levels were used for multiple* t*-tests. Data were expressed as median and interquartile range unless otherwise. Unmatched spots or spots with significantly different %V were considered “differently expressed” in the groups. Comparisons between differently expressed proteins as a function of *MECP2* mutation type were evaluated using either Mann-Whitney rank sum test or Kruskal-Wallis test. The effects of small population sizes on possible type I (*α*)/type II (*β*) errors in the data interpretation were examined using a sampling size algorithm. A two-sided *P* < 0.05 was considered to indicate statistical significance. The MedCalc version 12.1.4 statistical software (MedCalc Software, Mariakerke, Belgium) was used.

## 3. Results

### 3.1. Clinical Severity and *MECP2* Mutation Types

Median clinical severity score for the whole Rett population was 17 (inter-quartile range 9–21.2, values range 7–31).

### 3.2. Routine Haematology/Clinical Chemistry

**Figure 1:**
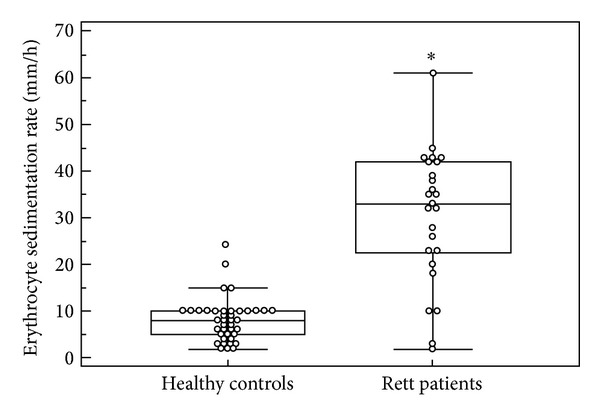
Erythrocyte sedimentation rate (ESR) measurements by TEST 1 in healthy controls and RTT patients. *P < 0.001.

Elevated ESR values (median 33.0 mm/h versus 8.0 mm/h, *P* < 0.0001) were detectable in RTT (Figure 1), whereas CRP levels were within the reference range (RTT median 0.04 mg/dL inter-quartile range 0.02–0.08 mg/Dl, values range 0.01–0.18 versus Control median 0.04 mg/dL inter-quartile range 0.001–0.11 mg/dL, values range 0.01–0.86, *P* = 0.6343). The existence and relevance of an unrecognized subclinical inflammatory status in Rett syndrome are strongly suggested by the evidence of relationships between ESR values and phenotypical severity of the disease (Figure 4).

### 3.3. Protein Expression Profile Differences Between RTT and Healthy Control Subjects

**Figure 2:**
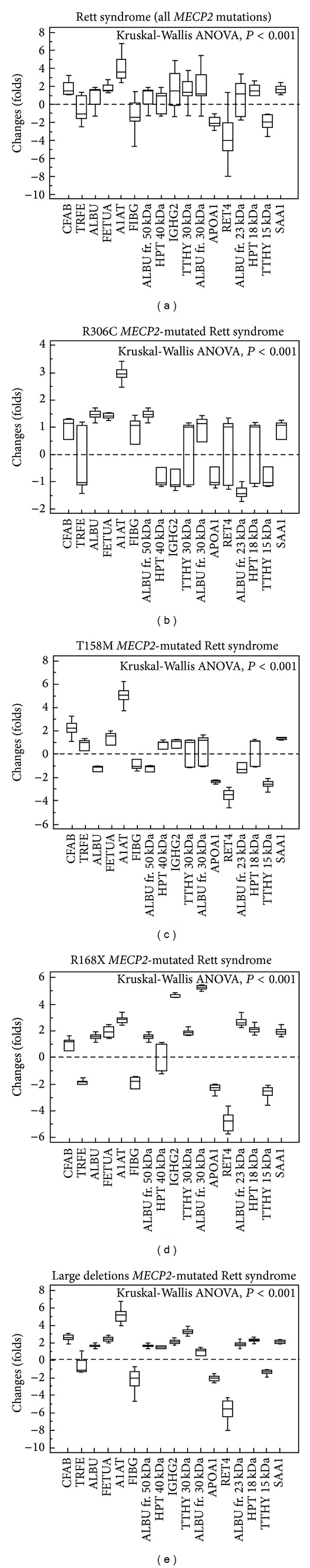
Plasma proteins expression as a function of *MECP2 *mutations in girls with classical Rett syndrome. (a) All *MECP2 *mutations, (b) R306C mutation (milder form), (c) T158M mutation (intermediate severity), (d) and (e) correspond to R168X and large deletions (severe forms), respectively. Data are compared to matched healthy controls and expressed as box-and-whiskers plots. Results of Kruskal-Wallis ANOVA are indicated.

**Figure 3:**
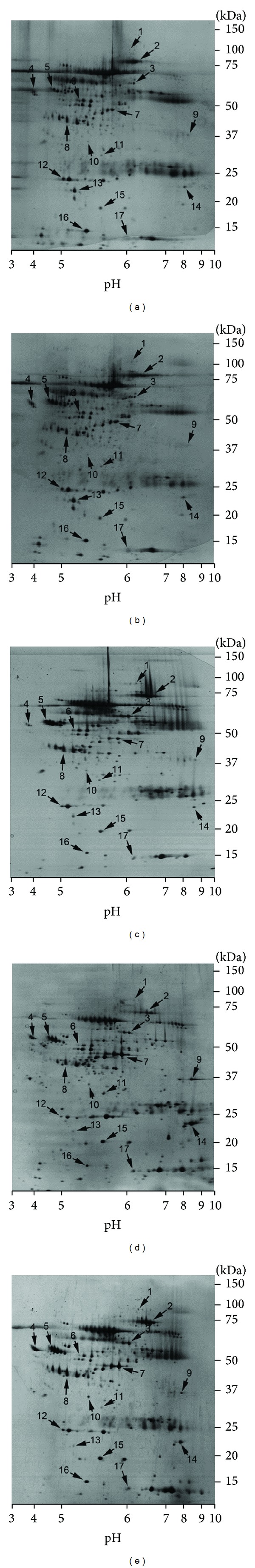
Silver-stained 2-DE gel of proteins from a typical healthy control (a), R306C (b), T158M (c), R168X (d), and large deletions (exons 3 and 4) (e). 60 *μ*g of total protein was subjected with nonlinear IPG strips, with a pH range of 3 to 10, followed by SDS-polyacrylamide gradient gel (8–16% T) electrophoresis. Numbers denote the identified proteins by mass spectrometry and are listed in Table 1 and Supplementary Table. Molecular mass (kDa) and pI markers are indicated along the gels.

**Figure 4:**
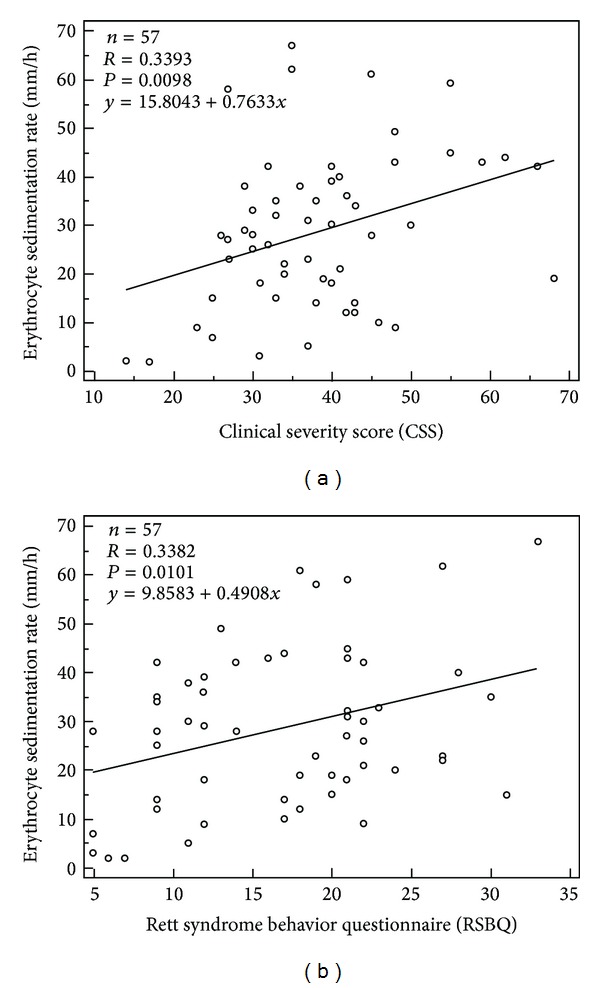
Statistically significant positive correlations were observed between erythrocytes sedimentation rate (ESR) and the clinical severity of the disease, as measured by (a) Clinical Severity Score (CSS) and (b) Rett Syndrome Behaviour Questionnaire (RSBQ).

Changes for a total of 17 different proteins were identified and expressed as folds changes relative to controls (Figures 2(a)–2(e) and see Supplementary Table in Supplementary Material available online at http://dx.doi.org/10.1155/2014/480980) and shown in 2-DE maps (Figures 3(a)–3(e)). Proteins were subsequently identified by mass spectrometry. Protein identification as well as peptide matches, sequence coverage, and the probabilistic score were obtained using the MASCOT software. The biological functions and their role in APR are summarized in Table 1. Actually, the majority of the proteins were categorized as either positive APR proteins (*n* = 6 spots, such as complement factor B (CFAB, spot 1), alpha-1-antitrypsin (A1AT, spot 5), fibrinogen gamma chain (FIBG, spot 6), haptoglobin (HPT, spots 8 and 15), and serum amyloid A-1 protein (SAA1, spot 17)) or negative APR proteins (*n* = 9 spots, such as serum transferrin (TRFE spot 2), albumin (ALBU, spot 3 as whole protein while spots 7, 11, and 14 as C terminal fragments), transthyretin (TTHY, spots 10 and 16), apolipoprotein A1 (APOA1, spot 12), and retinol-binding protein 4 (RET4, spot 13)) and, to a lesser extent, as proteins involved in the immune system (*n* = 2 spots, such as alpha-2-HS-glycoprotein (FETUA, spot 4) and Ig gamma-2 chain C region (IGHG2, spot 9)). Some proteins have known overlapping functions on metabolism and signal transduction (*n* = 10 spots, such as TRFE (spot 2), ALBU (spots 3, 7, 11, and 14), FIBG (spot 6), TTHY (spots 10 and 16), APOA1 (spot 12), and RET4 (spot 13)). The number of protein changes was found to be proportional to the severity of the *MECP2 *gene mutation.

SAA1, AIAT, and CFAB were all overexpressed in the examined *MECP2* mutation types. The behaviour of HPT appears to be discrepant among different *MECP2 *mutations RTT, with the 40 kDa spot being overexpressed in T158M and large deletions, while underexpressed in the R306C and normally expressed in the R168X mutation type. The HTP 18 kDa spot appears to be normally expressed in R306C and T158M while being overexpressed in the plasma samples from patients with R168X and large deletions. A1AT (spot 5) was significantly overexpressed (*P* < 0.001) in all *MECP2* mutation types and varied from +2.96 in R306C to +5.15 in large deletions when compared to controls (Figure 2). Furthermore, our data indicate that FETUA is overexpressed in all the *MECP2* mutations examined. On the other hand, RET4 and APOA1 were significantly underexpressed in three of the four examined *MECP2* gene mutation classes, that is, T158M, R168X, and large deletions. The TTHY 15 kDa spot (very close to the theoretical MW of 15,900 Da) was found to be underexpressed in all the examined mutation types and the TRFE spot appeared to be underexpressed in the R168X mutation type. A detailed analysis of protein expression patterns as a function of *MECP2* gene mutation types is reported as Supplementary Material (*Protein Expression Patterns in the Different MECP2 Mutation Types: Detailed Analysis).*

### 3.4. Protein-Protein Interaction Analysis

**Figure 5:**
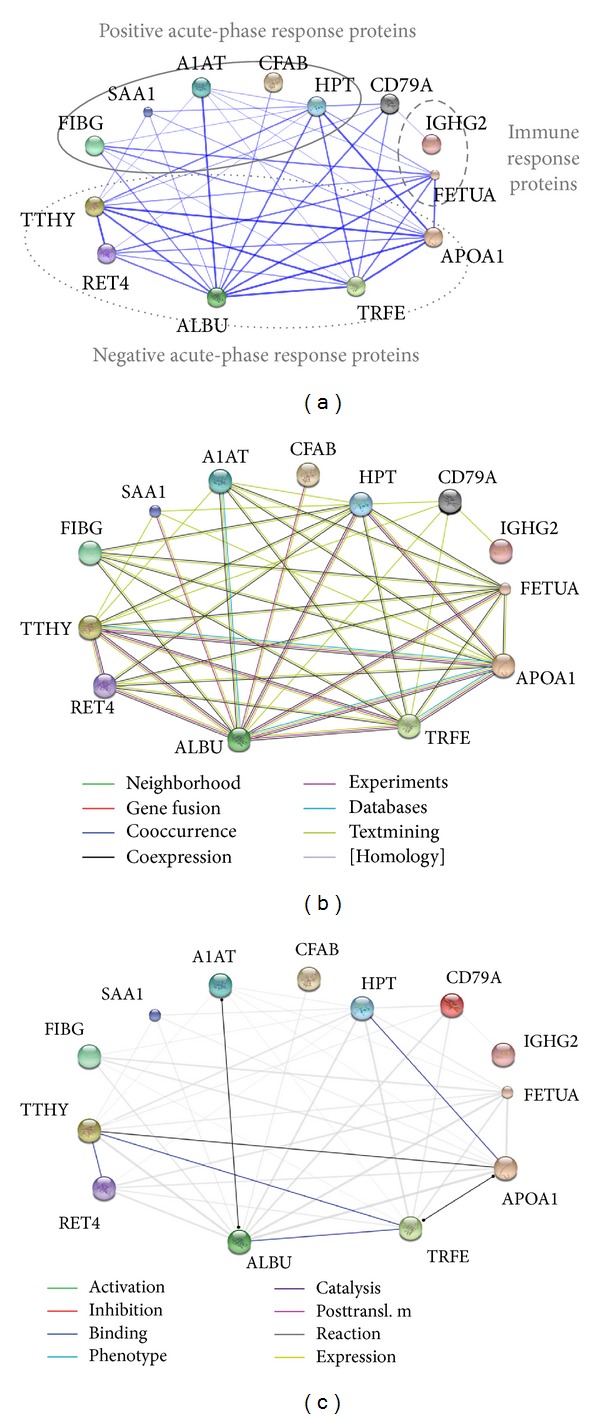
Predicted protein-protein interaction networks (PPI) created by STRING 9.05. Each circle represents an individual protein with the recognized abbreviated name. (a) Confidence PPI in which the thickness of the lines shows how strong the interactions are (threshold: 0.4, medium confidence). Positive, negative APR proteins and immune response proteins are highlighted in three different ellipsis. CD79A is represented with grey colour to indicate an unidentified protein by MS but helpful for linking IGHG2 with HPT. (b) Evidence PPI in which the colour of the lines represent the types of evidence which characterized the protein-protein association. Code lines: green: neighborhood, brown: coexpression, pink: experiments, sky-blue: databases, olive green: textmining. (c) Actions PPI of all proteins in which the different colour of the lines represent the mode of protein actions. Code lines: black: reaction, blue: binding.

In the RTT population identified proteins undergoing significant changes, potentially classifiable as positive APR proteins, negative APR proteins and immune response proteins, appear to be closely connected. STRING software created a prediction of protein-protein interaction networks (PPI) based on confidence (Figure 5(a)), evidence (Figure 5(b)) and actions (Figure 5(c)). In the confidence PPI, the thickness of the lines shows how strong the interactions are (threshold: 0.4, medium confidence). The strongest interactions were between TTHY and RET4 (combined association score: 0.997), APOA1 and ALBU (combined association score: 0.994), and TTHY and APOA1 (combined association score: 0.994) (Figure 5(a)). Positive APR proteins, negative APR proteins, and immune response proteins are highlighted in three different ellipses. In the evidence PPI, the colours of the lines represent the types of evidence which characterized the protein-protein association. More evidence was between TTHY and RET4 (combined association score: 0.997), ALBU and APOA1 (combined association score: 0.994), and TTHY and APOA1 (combined association score: 0.993) (Figure 5(b)). In the actions PPI, the different colours of the lines represent the mode of action of proteins. Code lines: black is reaction between TTHY and APOA1 (combined association score: 0.993), A1AT and ALBU (combined association score: 0.951), and APOA1 and TRFE (combined association score: 0.982) while blue is binding between TTHY and RET4 (combined association score: 0.997), TRFE and ALBU (combined association score: 0.988), HPT and APOA1 (combined association score: 0.975), and TTHY and TRFE (combined association score: 0.970) (Figure 5(c)).

## 4. Discussion

Etiology of autism is still unknown although several explanations have been proposed [35], including environmental factors combined to particular genotype [36], vaccines [37], and phthalates [38]. Experimental and clinical evidence accumulated in the last decades indicates the presence of an early immune activation in ASDs with an associated peripheral and central chronic inflammation [12–16]. In particular, a subclinical chronic inflammatory condition has been previously suggested to occur in autism [35].

Inflammation, as the first nonspecific defensive response in mammalians, is a component of the innate immunity, a self-protective process whose aim is to remove harmful stimuli in order to maintain the homeostasis [39]. Chronic inflammation differs from the acute inflammatory processes because of the duration, types of involved cells and primary mediators (i.e., cytokines, reactive oxygen species, hydrolytic enzymes, and growth factors versus eicosanoids and vasoactive amines), site (tissue versus vascular), and outcome [40]. Besides a few isolated studies regarding complement factors, TRFE and A1AT [41–43], not consistent information regarding a correlation between our individual identified plasma protein and ASDs or RTT exist in literature. Cumulating evidence indicates a link between astrocytes abnormalities and Rett syndrome [44–47]. In particular, a recent report analyzing the response of astrocytes during activation by proinflammatory stimulation [48] proposes that the protective phenotype against iron-mediated oxidative stress in cell death involves a complex change in the expression and activity of several genes involved in the control of the cellular redox state. Therefore, it is plausible that abnormal redox status and unrecognized pro-inflammatory stimuli would converge in RTT patients to functionally damage astrocytes.

The present study indicates, for the first time, the occurrence of a subclinical persistent inflammatory status in RTT patients with stage II (i.e., “pseudo-autistic”). In addition, our findings show a direct relationship between the number of the plasma protein quantitatively modified and the clinical severity of the disease.

A moderately increased ESR value was the only standard laboratory clue for an underlying inflammatory process, whereas, interestingly, other standard routine tests (i.e., CRP) appear to be unchanged. At the molecular level, production of CRP is induced by proinflammatory cytokines IL-1, IL-6, and IL-17 [49]. It is plausible that a cytokine dysregulation may exist in Rett syndrome, although no clear demonstration has been so far brought in this sense. A cytokine dysregulation can be inferred by an interesting parallel between RTT and APR protein variations in perinatal human plasma [50].

Plasma proteome analysis by 2-DE/MALDI-TOF is known to be able to identify even subtle changes with high specificity in protein identification and recognition of structural changes and posttranslational modifications [51]. This approach has allowed in the present study to unveil the upregulation of several positive APR proteins, such as SAA1 and A1AT [52, 53], as well as the downregulation of known negative APR proteins, such as APOA1 and RET4 [54, 55]. SAA1, A1AT, and CFAB, known as positive APR proteins, are all overexpressed in the examined *MECP2* mutation types. SAA1 is a major acute phase reactant and an apolipoprotein belonging to the HDL complex. Inflammatory cells chemotaxis, positive regulation of cytokines secretion, and platelet activation are some of its functions [52]. A1AT is a multifunctional protein involved in anti-inflammatory and tissue protective properties by protecting tissues from enzymes of inflammatory cells. More broadly, A1AT plays an important role in modulating immunity, inflammation, apoptosis, and possibly cellular senescence programs [53, 56]. By comparing R168X to T158M it is interesting to note that A1AT (spot 5) is underexpressed, whereas one C-terminal fragment of ALBU (spot 11) appears to be overexpressed, thus supporting the hypothesis that in the most severe RTT phenotypes more proteolysis occurs along with a lower activity by protease inhibitors, including A1AT. CFAB, a component of the alternate pathway of the complement system which contributes to generate C3 or C5 convertase and plays a role in the hemolytic uremia complex [57], appears to be overexpressed in T158M and large *MECP2* gene deletions. Nonetheless, none of the features of the hemolyticuremia complex is present in RTT.

Hence, we can suppose that the “pseudo-autistic” phase of RTT is characterized by a tissue damage to which an adaptive/defensive response ensues. To this regard, the mounting evidence of a persistent redox imbalance in RTT [6, 58] appears to be related to the wider context of a chronic inflammatory process whose fine mechanisms remain to be elucidated.

As predicted by the STRING software (Figure 5) the most strong interaction was represented by TTHY and RET4 (combined association score: 0.997) and demonstrated by the binding of RET4 to TTHY when circulating in plasma (in a 1 to 1 stoichiometry). In vitro one tetramer of TTHY can bind two molecules of RET4 [59].

TTHY has been suggested to be a transporter for thyroxin from the bloodstream to the brain [60]. To this regard, subtle changes in the levels of the thyroid hormones have been reported in RTT [61], although no evidence of clinical hypothyroidism is present in the affected patients.

TTHY shows in the proteomic maps, a contrasting behavior, with the 15 kDa spot being uniformly underexpressed in all the examined RTT patients, while the 30 kDa spot appears to be normally expressed in the R306C and T158M patients, but overexpressed in the R168X and *MECP2* large deletion mutation types. In the light of our findings, the observed underexpression of the TTHY 15 kDa spot could be interpreted as mirroring the behavior of a negative APR protein. The TTHY spot which appears to be corresponding to a higher MW (exactly 34,400 Da) already reported in the literature whose biological meaning is still to be clarified [62]. A likely source for the MW discrepancy could reside in the reducing experimental condition of 2-DE which could lead to splitting of the whole original protein into two subunits.

Another TTHY interaction was with APOA1 (combined association score: 0.994), demonstrated as relevant not only in physiological condition but also in amyloidosis (Reactome Pathways as of October 2012, visit http://www.reactome.org/ for the latest updates).

APOA1 and RET4 were found to be significantly underexpressed, as compared to controls, in three out of four examined *MECP2* gene mutations (i.e., T158 M, R168X, and large deletions). Both proteins are involved in the lipid metabolism and have been suggested to be involved in the dyslipidemia of children with metabolic syndrome [54, 55, 63]. On the other hand, their involvement in RTT appears to support the concept of an altered lipid metabolism in this condition featuring hyperleptinemia [64] and hypercholesterolemia [65] and for which the coexistence of a “fatty acids paradox” has been suggested, given that an excessive endogenous fatty acids oxidation is paradoxically ameliorated by administration of the same exogenous fatty acids [66, 67].

Interestingly, also the interaction of APOA1 with ALBU (combined association score: 0.994) takes place in the lipid metabolism particularly in the pathway of HDL-mediated lipid transport.

In contrast, the interaction of APOA1 with TRFE (combined association score: 0.993) is involved in the release of platelet secretory granule components (Reactome Pathways as of October 2012, visit http://www.reactome.org/ for the latest updates).

In our findings, TRFE was underexpressed in the R168X mutation type, a variation which is in accordance with the classical negative APR protein changes.

In another biochemical pathway, HPT can bind APOA1 and impairs its stimulation of lecithin:cholesterol acyltransferase (LCAT). LCAT plays a major role in reverse cholesterol transport [68]. HPT is a protein which captures and combines with free plasma hemoglobin to allow hepatic recycling of heme iron in order to prevent kidney damage [69, 70]. Its behaviour appears to be discrepant among different *MECP2 *mutations RTT, with the 40 kDa spot being overexpressed in T158M and large deletions, while underexpressed in the R306C and normally expressed in the R168X mutation type. The HTP 18 kDa spot appears to be normally expressed in R306C and T158M while being overexpressed in the most severe examined mutations (i.e., R168X and large deletions).

Significant changes in RTT plasma were evidenced in FETUA and IGHG2 both involved in the immune response. In particular, FETUA is directly connected with APOA1 (combined association score: 0.874). Our data indicate that FETUA is overexpressed in all the *MECP2* mutations examined. FETUA is synthesized in the liver and subsequently concentrated in bone matrix. This protein is known to promote endocytosis, possess opsonic properties, and influence the mineral phase of bone. FETUA shows high affinity for calcium and barium ions [71]. To this regard, cumulating evidence indicates that osteoporosis is a phenotypic feature of RTT [72, 73].

Of course, several unsolved questions arise from the present study, including the causes of APR in the autistic phase of RTT. A stimulating parallel is the recent demonstration of a subclinical inflammatory process in autism [35]. Therefore, inflammation seems to be a previously unrecognized feature in autistic and cognitive disorders, which could play a role in the evolution of the pathology and modulations of phenotypical severity.

## 5. Conclusion

Cumulating evidence indicates that RTT is a multisystemic disorder, with the involvement of lung [53], bone [72, 73], heart [74], and gastrointestinal apparatus [75, 76], besides the central nervous system. For the first time, we evidenced a subclinical chronic inflammatory status related to the severity carried by *MECP2* gene mutations in the “pseudo-autistic” (stage II) phase of RTT. Our detection of a persistent inflammatory status is compatible with a systemic disease and adds a new perspective in the pathogenesis and future therapeutic strategies for RTT [67] and ASDs.

## Supplementary Material

Details for differentially expressed plasma proteins in Rett Syndrome and controls.

## Figures and Tables

**Table 1:** Summary of the proteins identified as differently expressed using the proteomics approach.

Spot	SwissProt code	Protein name	Short name	Theoretical pI/Mr (kDa)	Peptides matches	Sequence coverage (%)	MOWSE score	Biological functions	APR proteins
1^a^	P00751	Complement Factor B	CFAB	6.67/86.8	22/39	37	219	Immune system, complement system regulation	(+)
2	P02787	Serum transferrin	TRFE	6.81/79.2	40/80	50	340	Iron binding and transport	(−)
3	P02768	Albumin	ALBU	5.92/71.3	30/62	55	268	Transport, regulation of colloidal osmotic pressure, platelet activation	(−)
4	P02765	Alpha-2-HS-glycoprotein	FETUA	5.43/40.09	10/22	38	110	Endocitosis, opsonization	N.A.^b^
5	P01009	Alpha-1-antitrypsin	A1AT	5.37/46.8	16/37	46	164	Acute phase response, coagulation, proteases inhibition	(+)
6	P02679	Fibrinogen gamma chain	FIBG	5.37/52.1	13/43	41	122	Coagulation, signal transduction	(+)
7	P02768	Albumin (C terminal fragment)	ALBU	5.92/71.3	16/29	28	138	Transport, regulation of colloidal osmotic pressure, platelet activation	(−)
8	P00738	Haptoglobin	HPT	6.13/45.8	6/10	14	51	Acute phase response, hemoglobin binding	(+)
9	P01859	Ig gamma-2 chain C region	IGHG2	7.66/36.5	5/8	13	71	Innate immunity	N.A.^b^
10	P02766	Transthyretin	TTHY	5.52/15.9	7/18	68	115	Thyroid hormone binding and transport	(−)
11	P02768	Albumin (C terminal fragment)	ALBU	5.92/71.3	18/27	28	188	Transport, regulation of colloidal osmotic pressure, platelet activation	(−)
12	P02647	Apolipoprotein A1	APOA1	5.56/30.7	26/87	65	213	Lipid transport and metabolism	(−)
13	P02753	Retinol-binding protein 4	RET4	5.76/23.3	8/18	57	104	Retinol transport and metabolism	(−)
14	P02768	Albumin (C terminal fragment)	ALBU	5.92/71.3	6/12	12	66	Transport, regulation of colloidal osmotic pressure, platelet activation	(−)
15	P00738	Haptoglobin	HPT	6.13/45.8	8/24	20	86	Acute phase response, hemoglobin binding	(+)
16	P02766	Transthyretin	TTHY	5.52/15.9	6/13	68	104	Thyroid hormone binding and transport	(−)
17	P0DJI8	Serum amyloid A-1 protein	SAA1	6.28/13.5	5/12	51	62	Acute phase response, apolipoprotein of the HDL complex	(+)

^a^Spot numbers match those reported in the representative 2-DE images shown in Figure 3. N.A.: not applicable. (+) and (−) indicate positive and negative APR proteins, respectively. ^b^Identified proteins showing variations in RTT for which a possible involvement in the inflammatory response is unknown.

## Abbreviations

CFAB: Complement factor B
TRFE: Serum transferrin
ALBU: Albumin
FETUA: Alpha-2-HS glycoprotein
A1AT: Alpha-1-antitrypsin
FIBG: Fibrinogen gamma chain
HPT: Haptoglobin
IGHG2: Ig gamma-2 chain C region
TTHY: Transthyretin
APOA1: Apolipoprotein A1
RET4: Retinol-binding protein 4
SAA1: Serum amyloid A-1 protein.
