# Supplementary material for: Subclinical Inflammatory Status in Rett Syndrome
Source: Mediators Inflamm. 2014 Jan 6;2014:480980. doi: 10.1155/2014/480980 (PMC3913335; doi:10.1155/2014/480980)
Supplement: Supplementary file 1 — Details for differentially expressed plasma proteins in Rett Syndrome and controls. [file 480980.f1.pdf]

Supplementary Table 1: Details for differentially expressed plasma proteins in Rett Syndrome and controls.

Protein						*MECP2* gene mutation type													
ID	Name	Control A	RTT		Control B	R306C		T158M			R168X				Large deletions (exons 3 and 4)				
		mean±SD	mean±SD	Trend A	mean±SD	mean±SD	Trend B	mean±SD	Trend B	Trend C	mean±SD	Trend B	Trend C	Trend D	mean±SD	Trend B	Trend C	Trend D	Trend E
1	CFBA	0.93±0.65	1.46±0.63	↑*	0.83±0.11	0.94±0.17		1.84±0.73	↑*	↑*	0.99±0.27			↓*	2.09±0.37	↑*	↑*		↑*
2	TRFE	4.06±0.62	3.57±0.85		4.02±0.53	3.92±0.83		4.13±1.22			2.14±0.41	↓*	↓*	↓*	4.10±0.92				↑*
3	ALBU	3.09±1.89	5.16±1.09	↑*	3.19±0.44	3.35±0.51		5.81±0.21	↑**	↑**	5.60±0.21	↑**	↑**		5.88±0.42	↑**	↑**		
4	FETUA	1.45±0.82	2.54±0.62	↑**	1.42±0.69	2.19±1.13		2.10±0.77			2.77±0.48	↑*			3.11±0.55	↑**	↑*	↑*	
5	A1AT	2.66±0.83	5.25±1.70	↑**	1.37±0.68	3.89±0.51	↑*	6.61±1.32	↑**	↑*	3.74±0.53	↑*		↓*	6.76±1.46	↑**	↑*		↑*
6	FIBG	1.28±0.50	1.00±0.43		1.31±0.56	1.38±0.45		1.25±0.31			0.72±0.16	↓*	↓*	↓*	0.68±0.11	↓*	↓*	↓*	
7	ALBU	4.85±2.01	7.08±1.93	↑*	4.17±0.32	7.59±1.16	↑*	4.31±0.41		↓*	8.18±1.45	↑**	↑*		8.23±1.55	↑**	↑*	↑**	
8	HPT	5.59±1.36	6.52±1.21	↑*	6.06±0.98	5.86±0.81		6.13±1.05			6.01±0.79				8.08±1.09	↑*	↑*	↑*	↑*
9	IGHG2	1.33±0.46	2.70±1.86	↑*	1.23±0.10	1.09±0.12		1.36±0.20			5.73±0.22	↑**	↑**	↑**	2.65±0.62	↑*	↑*	↑*	↓*
10	TTHY	0.95±0.60	1.82±0.89	↑*	0.99±0.14	0.97±0.12		1.03±0.16			1.86±0.31	↑*	↑*	↑*	3.19±0.44	↑**	↑**	↑**	↑*
11	ALBU	1.14±0.41	2.24±1.88	↑*	1.04±0.21	1.17±0.29		1.21±0.33			5.47±0.21	↑**	↑**	↑**	1.13±0.27				↓**
12	APOA1	4.78±2.22	3.18±1.28	↓*	5.40±0.26	5.24±0.77		2.48±0.45	↓*	↓*	2.38±0.35	↓**	↓**		2.63±0.57	↓*	↓*		
13	RET4	6.40±1.45	2.58±1.95	↓**	6.59±0.65	6.84±1.59		1.88±0.37	↓*	↓*	1.39±0.29	↓**	↓**		1.21±0.31	↓**	↓**		
14	ALBU	2.79±0.62	4.85±2.80	↑*	3.05±0.41	2.3±0.49		2.42±0.62			8.78±1.56	↑**	↑**	↑**	5.92±0.45	↑*	↑*	↑*	
15	HPT	3.07±0.54	5.58±2.15	↑**	3.51±0.23	3.5±0.42		3.71±0.60			7.46±1.31	↑**	↑**	↑**	7.64±1.30	↑**	↑**	↑**	
16	TTHY	6.59±1.29	5.08±2.19	↓*	8.03±1.24	7.87±1.31		3.21±0.58	↓**	↓**	3.18±0.69	↓*	↓*		6.04±1.46			↑*	↑*
17	SAA1	1.26±0.22	2.07±0.57	↑*	1.15±0.11	1.38±0.14		1.87±0.32			2.47±0.48	↑*	↑*		2.58±0.37	↑*	↑*		

↓ protein spot underexpressed; ↑ protein spot overexpressed; - protein spot disappearance; + protein spot appearance; N.D. not detectable. A1AT, Alpha-1-antitrypsin; AMBP, Alpha-1-microglobulin; ALBU, Albumin; CLUS, Clusterin; CO3, Complement C3; FIBB, Fibrinogen beta-chain; HBB, Hemoglobin subunit beta; HPT, Haptoglobin; IGHG2, Immunoglobulin gamma-2 chain C region; IGJ, Immunoglobulin J chain; TRFE, Serum transferrin; TTHY, Transthyretin. Numbers in the parentheses indicated the number of patients or subjects who are compared.
